# Supplementary material for: Temporal inhibition of autophagy reveals segmental reversal of ageing with increased cancer risk
Source: Nat Commun. 2020 Jan 16;11:307. doi: 10.1038/s41467-019-14187-x (PMC6965206; doi:10.1038/s41467-019-14187-x)
Supplement: Supplementary file 1 — Supplementary Information [file 41467_2019_14187_MOESM1_ESM.pdf]

Supplementary Information

Temporal inhibition of autophagy reveals segmental reversal of ageing with increased cancer risk

Cassidy et al.

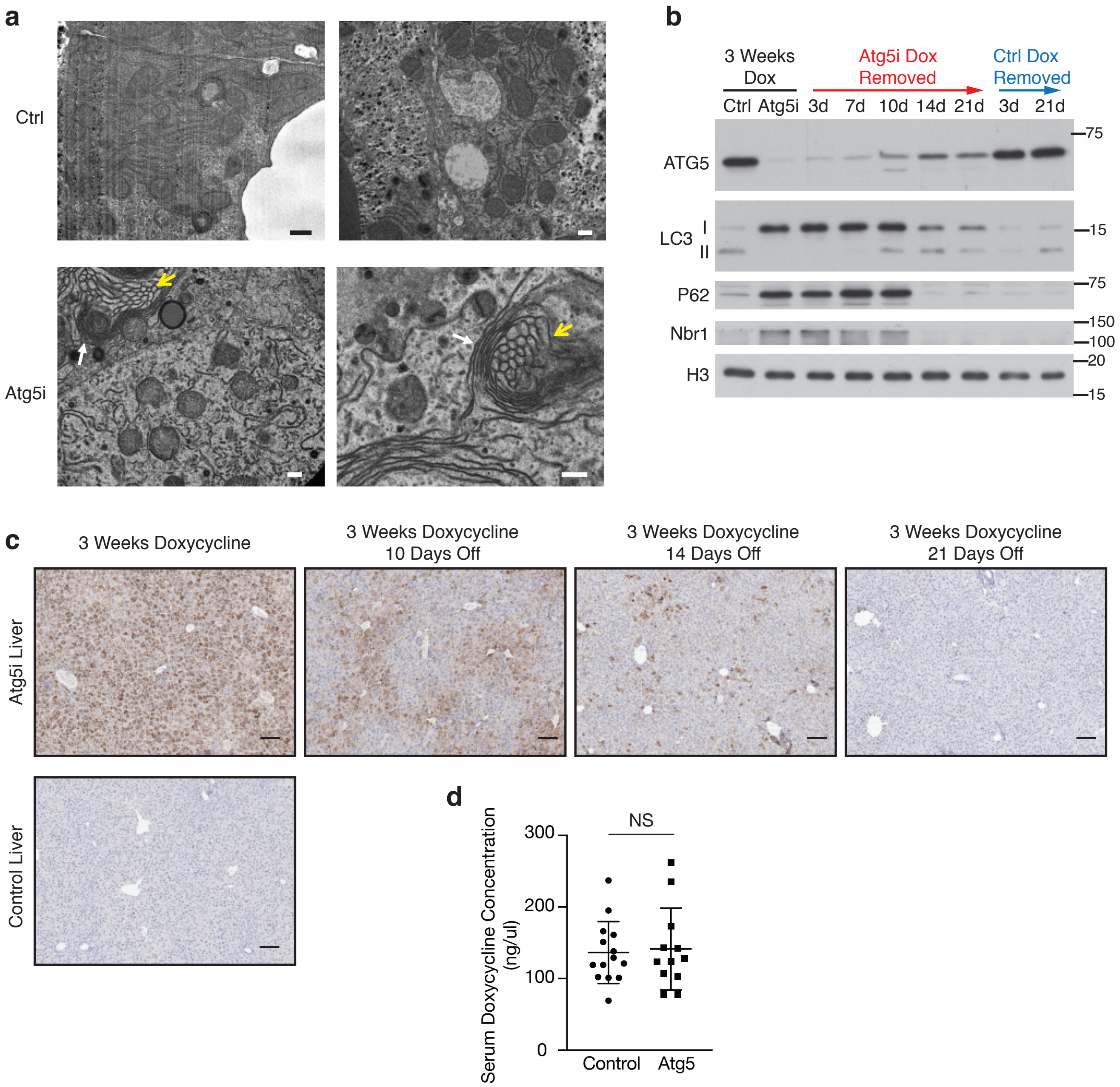

Supplementary Figure 1: Characterisation of Atg5i mice

a, Livers from Atg5i mice treated with doxycycline for 6 weeks display evidence of stacked (white arrow) and vacuolated (yellow arrow) membranes, not seen in control mice. Scale bars, 500 nm. **b**, Atg5i mice enable a dynamic control of autophagy as shown through a flux experiment. Briefly mice were given a doxycycline containing diet for 3 weeks, before being placed onto a diet absence of doxycycline for 3 weeks. Liver from autophagy inhibited mice display a dramatic reduction in Atg5 and an increase in LC3-I and Nbr1. Upon doxycycline removal Atg5 levels begin to recover at 10-14 days, a timepoint that coincides with the re-establishment of LC3-II. **c**,Similar data can be seen for p62 IHC. p62 levels in LT-Atg5i mice are evelated after 3 weeks of doxycycline treatment, in comparison to age-matched controls, before returning to baseline after a 3 week period. Scale bars,100 μm. **d**, Steady-state serum doxycycline levels are the same between LT-Ctrl and LT-Atg5i mice treated with doxycycline for 4 months. Error bars indicate standard deviation; NS denotes not significant. For Supplementary Figure 1b source data are provided as a Source Data file.

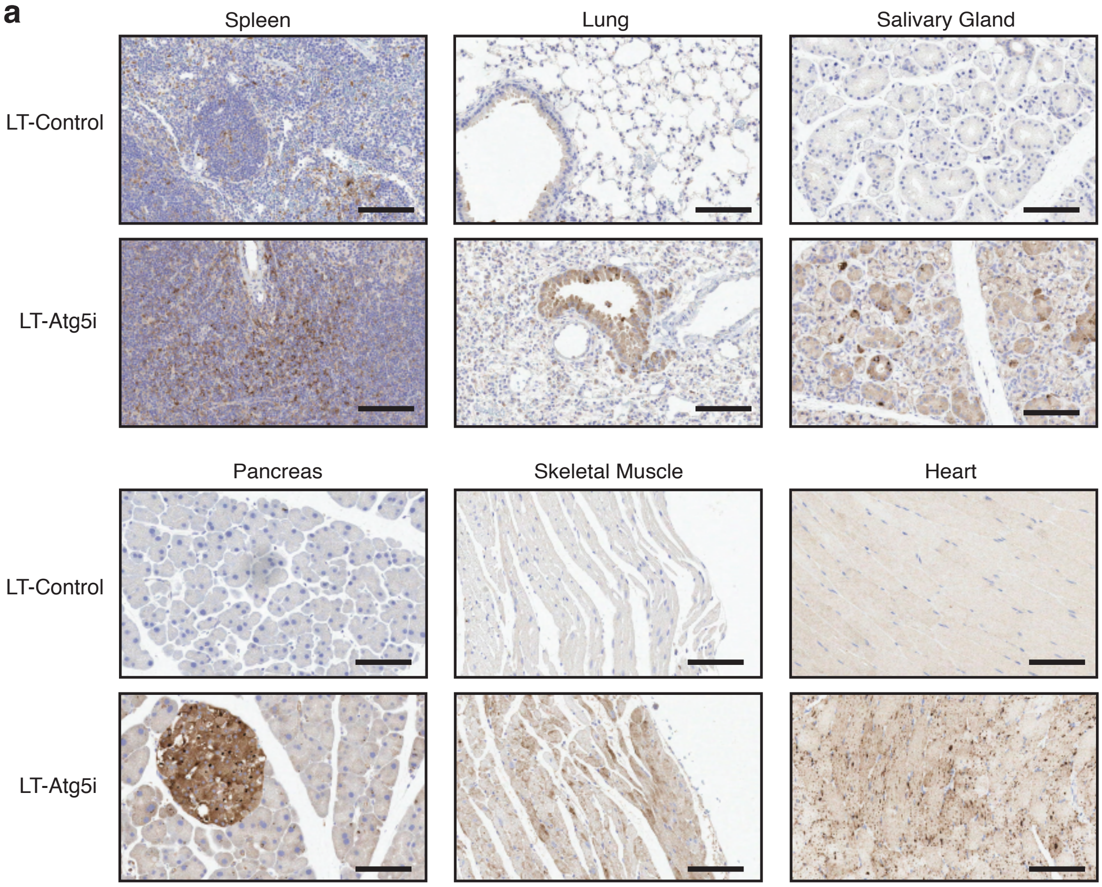

Supplementary Figure 2: p62 build-up in LT-Atg5i mice
a, As expected p62 levels in LT-Atg5i mice across numerous tissues are evelated after 4 months of doxycycline treatment, in comparison to age-matched controls.

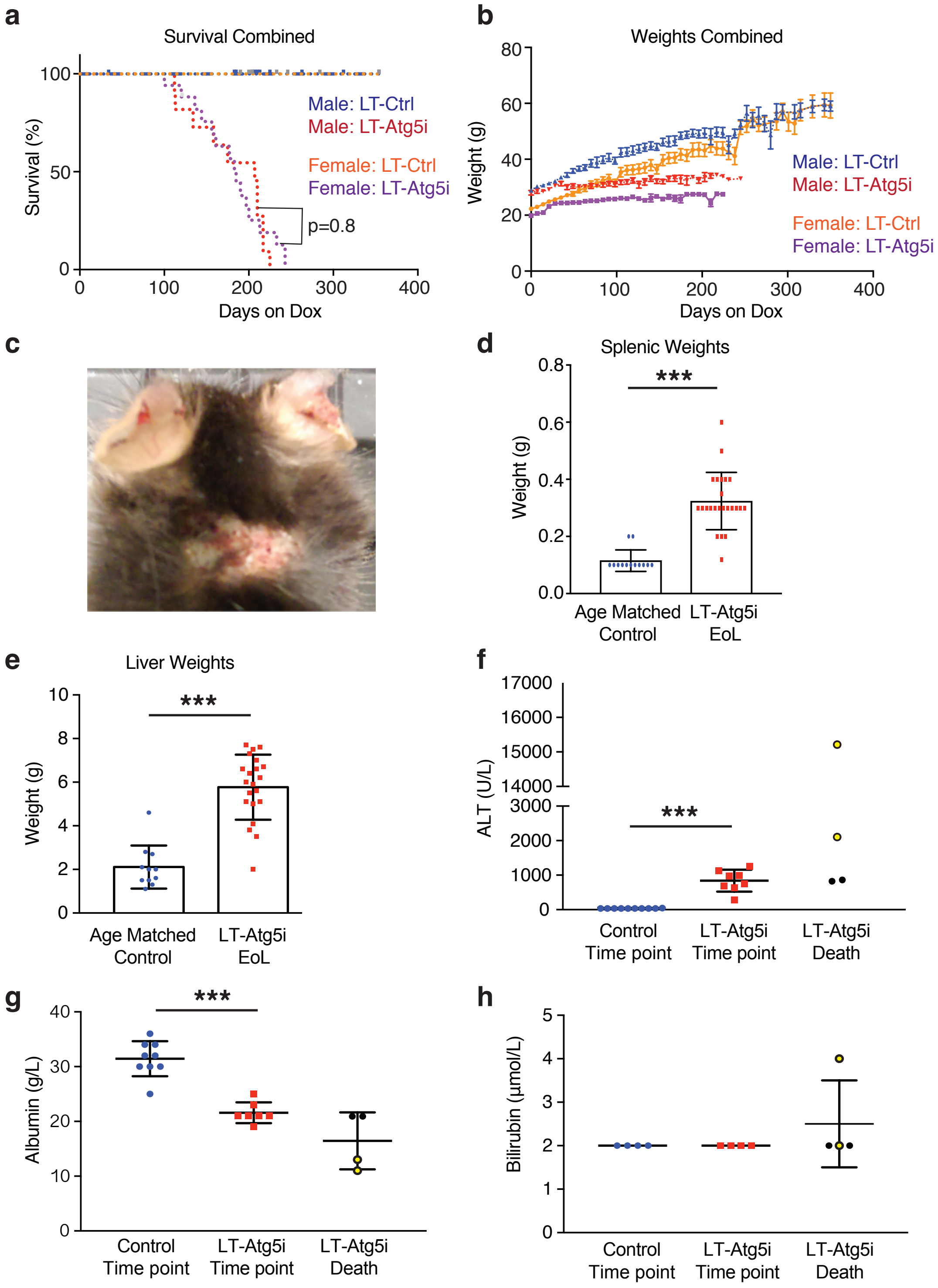

Supplementary Figure 3: Characterisation of LT-Atg5i mice
a, LT-Atg5i mice display no life-span associated sex bias (Red, LT-Atg5i Males; Purple, LT-Atg5i Females; p=0.8). **b**, LT-Atg5i mouse weight plateau while LT-Control mice continue to gain weight over their lifetime. **c**, Example of mouse suffering from ulcerative dermatitis. **d**, Splenic weights were increased in LT-Atg5i mice in comparison to age matched LT-Control mice. **e**, LT-Atg5i mice also display an increase in liver weight. **f-h**, liver function of LT-Atg5i mice as determined using serum samples. LT-Atg5i mice on dox for 4 months display an increase in serum ALT (**f**) and a decrease in serum albumin (**g**), that is further exacerbated in a subset of LT-Atg5i EoL (End of Life) individuals (yellow circles). The only sample tested that displayed an increase in serum bilirubin levels was also from a mouse displaying high levels of serum ALT and low levels of serum album (**h**). Error bars indicate standard deviations. *p<0.05; **p<0.01, ***p<0.001

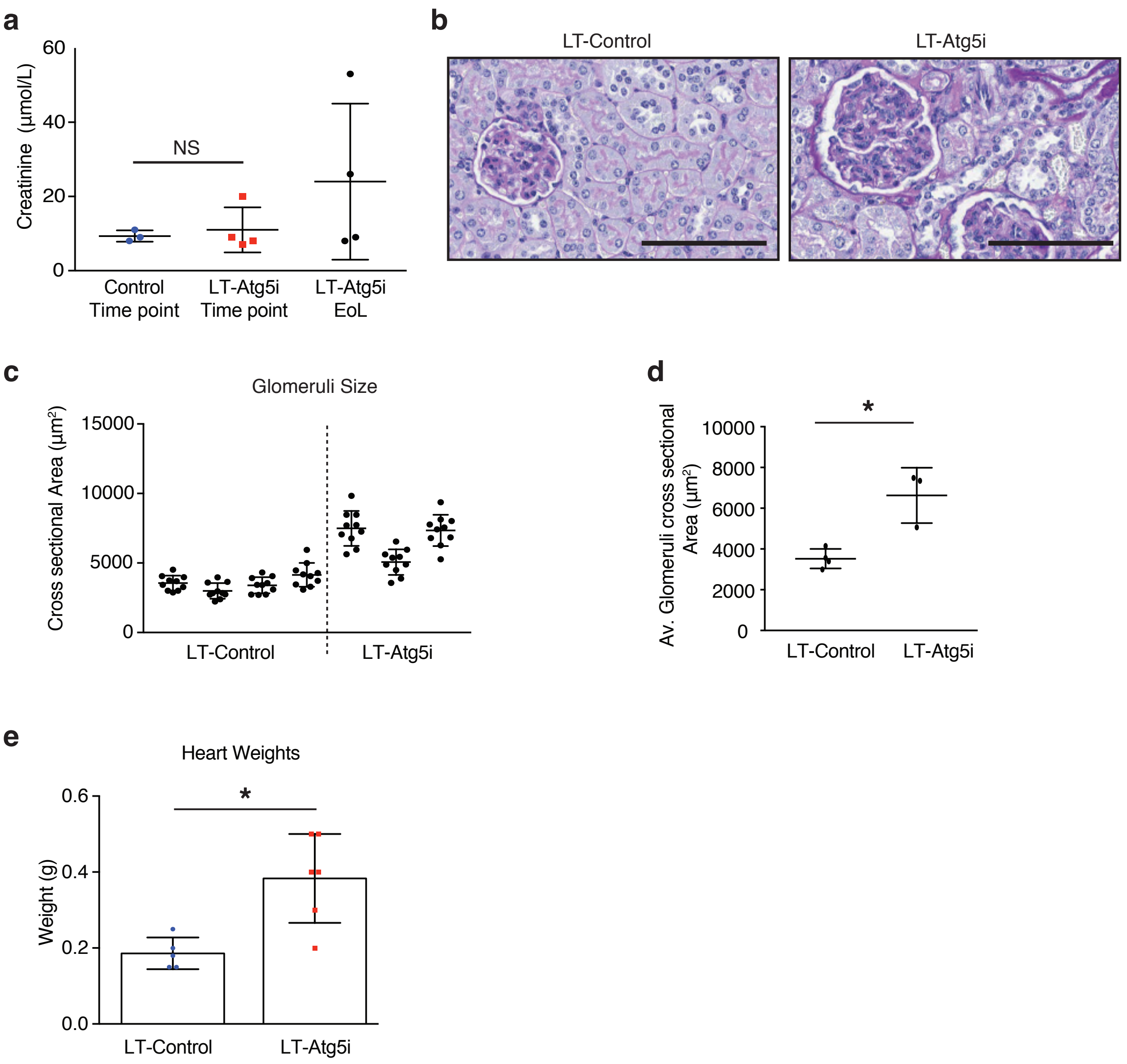

Supplementary Figure 4: Kidney and heart alterations in LT-Atg5i mice

a, LT-cohorts treated with doxycycline for 6 months mice display no significant differences in serum creatinine levels (unpaired two-tailed Welches t-test, NS denotes not significant; n= 3 LT-Control and 4 LT-Atg5i). At death, only a subset of LT-Atg5i mice display an increase in serum creatinine levels. **b-d**, LT-Atg5i mouse kidneys treated with doxycycline for 6 months present with evidence of sclerotic glomeruli determined using PAS stain that are also enlarged and hypercelluar in comparison to LT-Control (p=0.0479, unpaired two-tailed t-test; n= 4 LT-Control and 3 LT-Atg5i, the cross-sectional area of 10 randomly chosen glomeruli were measured per mouse). **e**, Cardiac tissue from LT-Atg5i mice at death was significantly heavier than age-matched LT-Control mice. (p=0.0108). Error bars indicate standard deviations. *p<0.05; **p<0.01, ***p<0.001. NS denotes not significant.

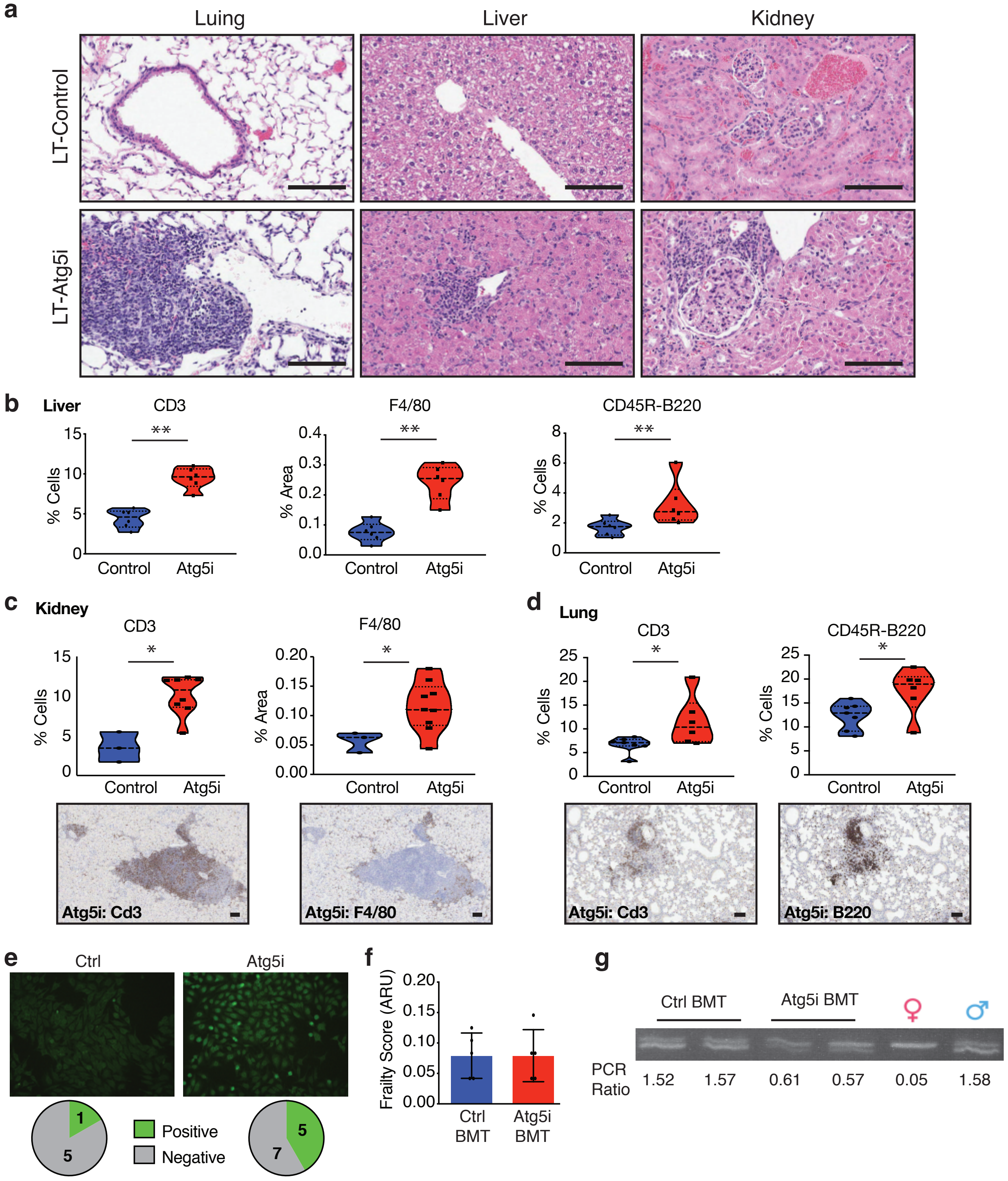

Supplementary Figure 5: Immune alterations in LT-Atg5i mice

a, LT-Atg5i mice display evidence of widespread immune infiltration across multiple tissues in comparison to age-matched controls. Scale bars,100 μm. **b-d,** Analysis of the composition of the immune infiltrate can be seen for liver (**b**), kidney (**c**), and lungs (**d**) (two-tailed Mann-Whitney test, between n=3-9 per group). **e**, Results from an Anti-Nuclear Antibody test of mouse serum samples after 4 months of doxycycline treatment. LT-Atg5i mice displayed an increased frequency of autoimmunity in comparison to age-matched controls. **f**, Frailty scores from irradiated C57BL/6 mice reconstituted with Ctrl or Atg5i bone marrow and treated with doxycycline for 4 months show no difference between conditions (unpaired two-tailed Welches t-test, between n=5 per group). **g**, PCR based analysis of chimerism in peripheral blood based on the ratio of PCR band intensity. All bone marrow donors were male and all recipients female. Only Atg5i bone marrow recipients shows a reduced ratio suggestive of reduced chimerism. Error bars indicate standard deviation.

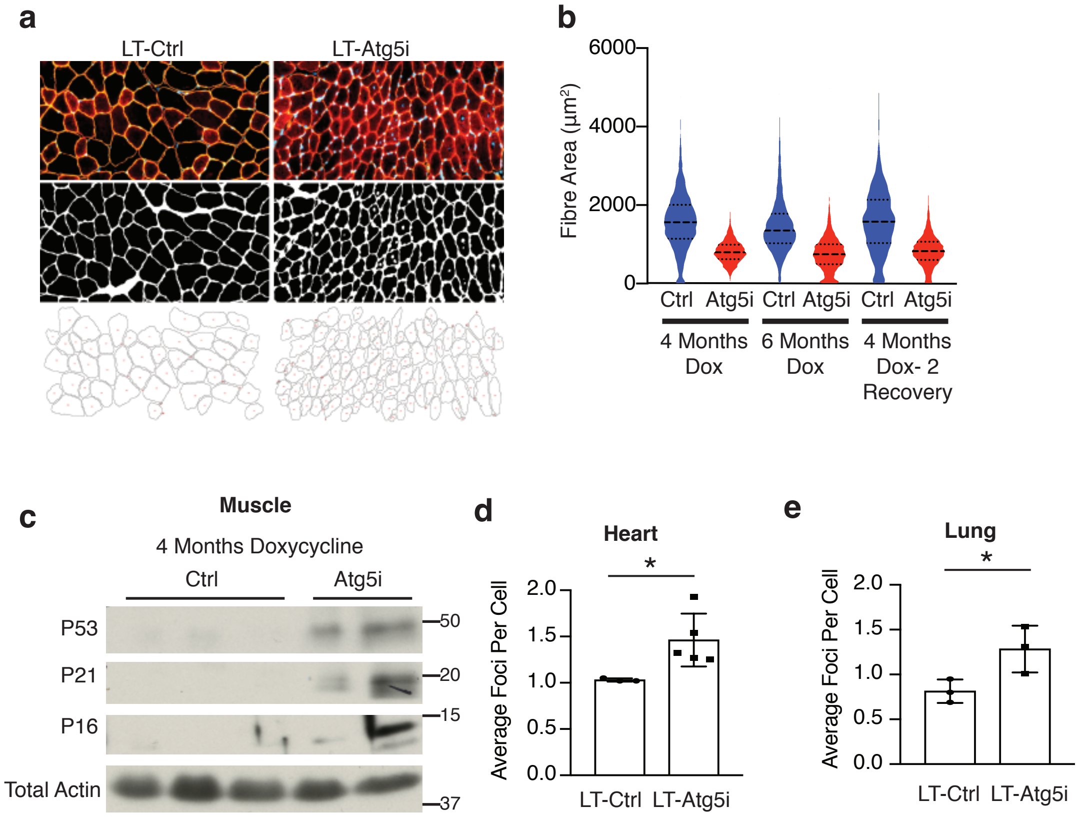

Supplementary Figure 6: Muscle and senescence phenotyping Analysis
a-c, Analysis of the muscle from LT-Atg5i mice. Example images of staining and morphometry analysis from muscle sections (**a**). Combined raw data of muscle fibre area for LT-cohorts treated with doxycycline for 4 and 6 months, as well as R-cohorts treated for 4 months and left 2 months to recover without doxycycline (**b**). LT-Atg5i muscle display evidence of senescence markers (**c**). **d**, TAF in heart and **e**, liver are increased in LT-Atg5i mice. (unpaired two-tailed t-test). Error bars indicate standard deviation; NS denotes not significant. *p<0.05; **p<0.01, ***p<0.001. For Supplementary Figure 6c source data are provided as a Source Data file.

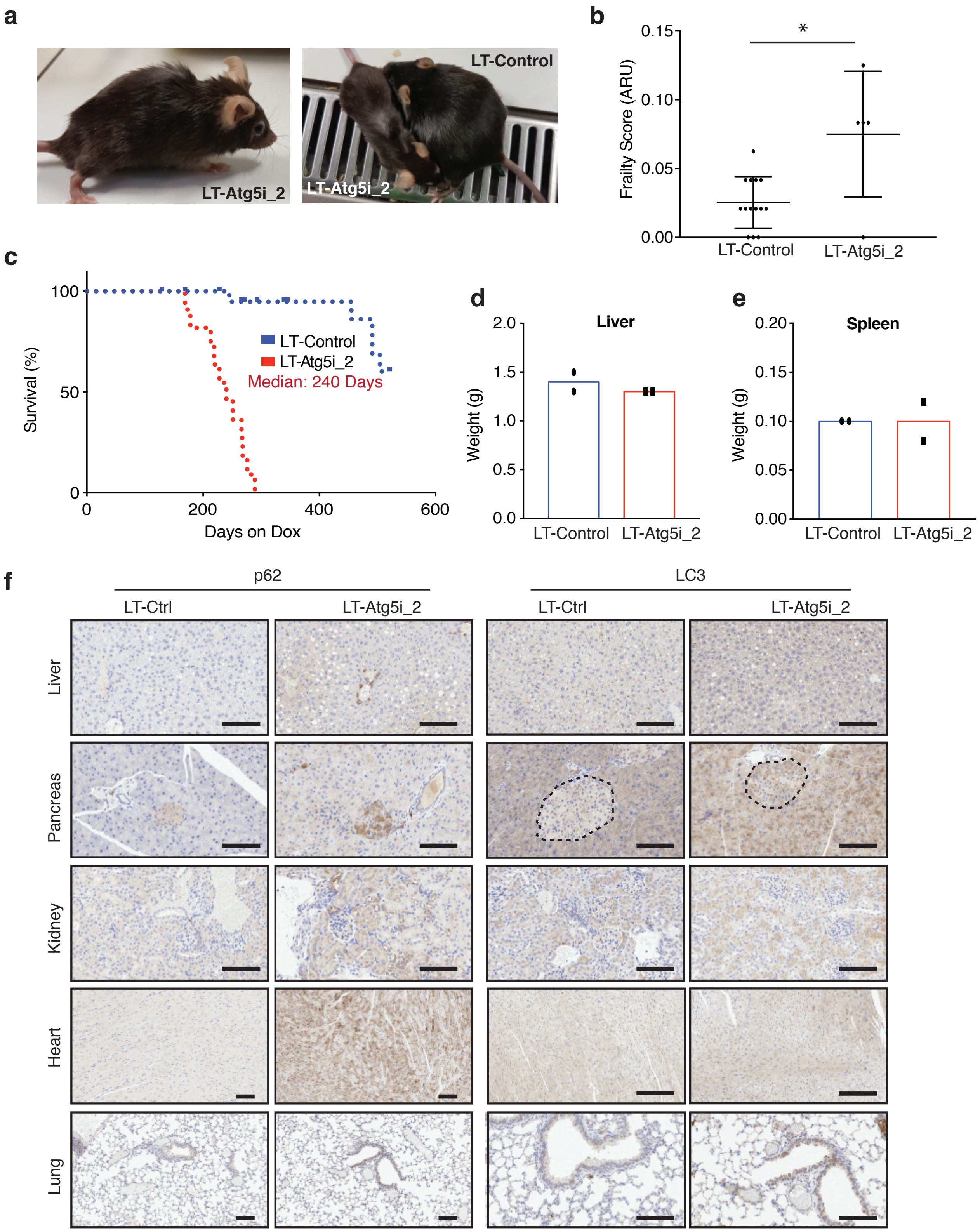

Supplementary Figure 7: Hypomorphic LT-Atg5i_2 mice also display aging phenotypes
a-c, LT-Atg5i_2 mice phenotypically recapitulate premature ageing phenotypes including kyphosis (**a**), increased frailty (**b**) (ARU, arbitrary units; Mann-whitney n= 14 LT-Control and 5 LT-Atg5i_2 mice), and reduced longevity (**c**). **d-f** However, Atg5i_2 mice appear to have a hypomorphic phenotype and do not recapitulate the phenotypes found in Atg5 knock-out and LT-Atg5i. These include no evidence of hepatomegaly (**d**) or splenomegaly (**e**). Correspondingly, p62/SQSTM1 and LC3 levels do not accumulate to the same degree in LT-Atg5_2 mice treated with doxycycline for 6 weeks (**f**). Scale bars,100 µm. Error bars indicate standard deviations. *$p<0.05$

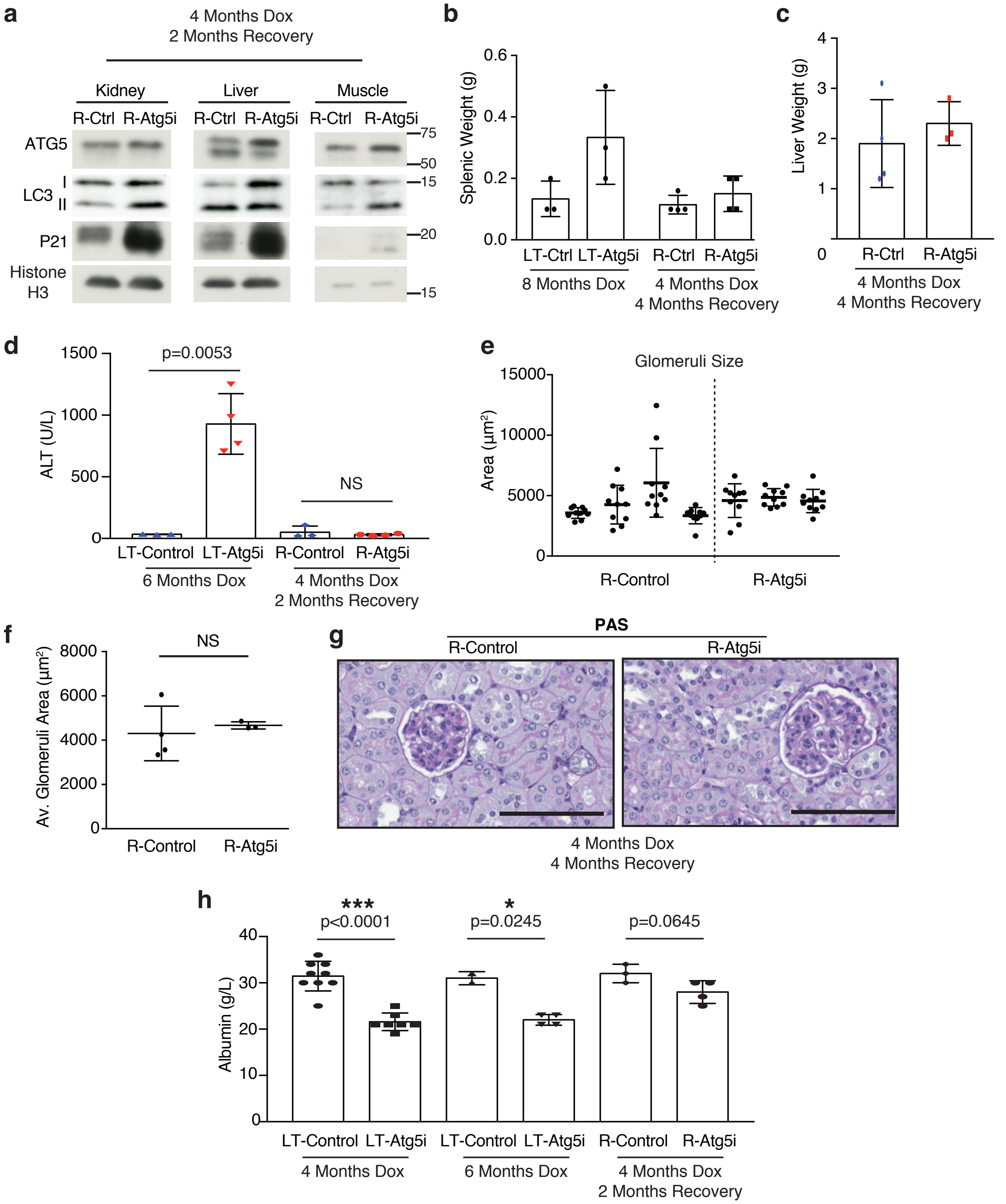

Supplementary Figure 8: Autophagy restoration reverses hepatomegaly and splenomegaly
a, Doxycycline removal is associated with an increase in Atg5 and restoration of Lc3 levels. **b**, Splenic and **c**, liver weights from R-Atg5i mice exhibit evidence of recovery. **d**, In addition, R-Atg5i mice display a reduction in serum ALT levels (unpaired two-tailed Welches t-test; n= 3-4 per cohort). **e**-**g**, R-Atg5i mice 4 months post dox removal display evidence of recovery in the kidneys as determined by (**e**-**f**) normalisation of glomeruli size appeared relative to age-matched controls (unpaired two-tailed Mann whitney, n=3-4 mice per group) and the absence of sclerosis (**g**). **h**, A partial recovery in serum albumin levels is also present in these mice unpaired two-tailed Welches t-test; n= 2-9 per cohort). Error bars indicate standard deviation; NS denotes not significant. *p<0.05; **p<0.01, ***p<0.001. For Supplementary Figure 8a source data are provided as a Source Data file.

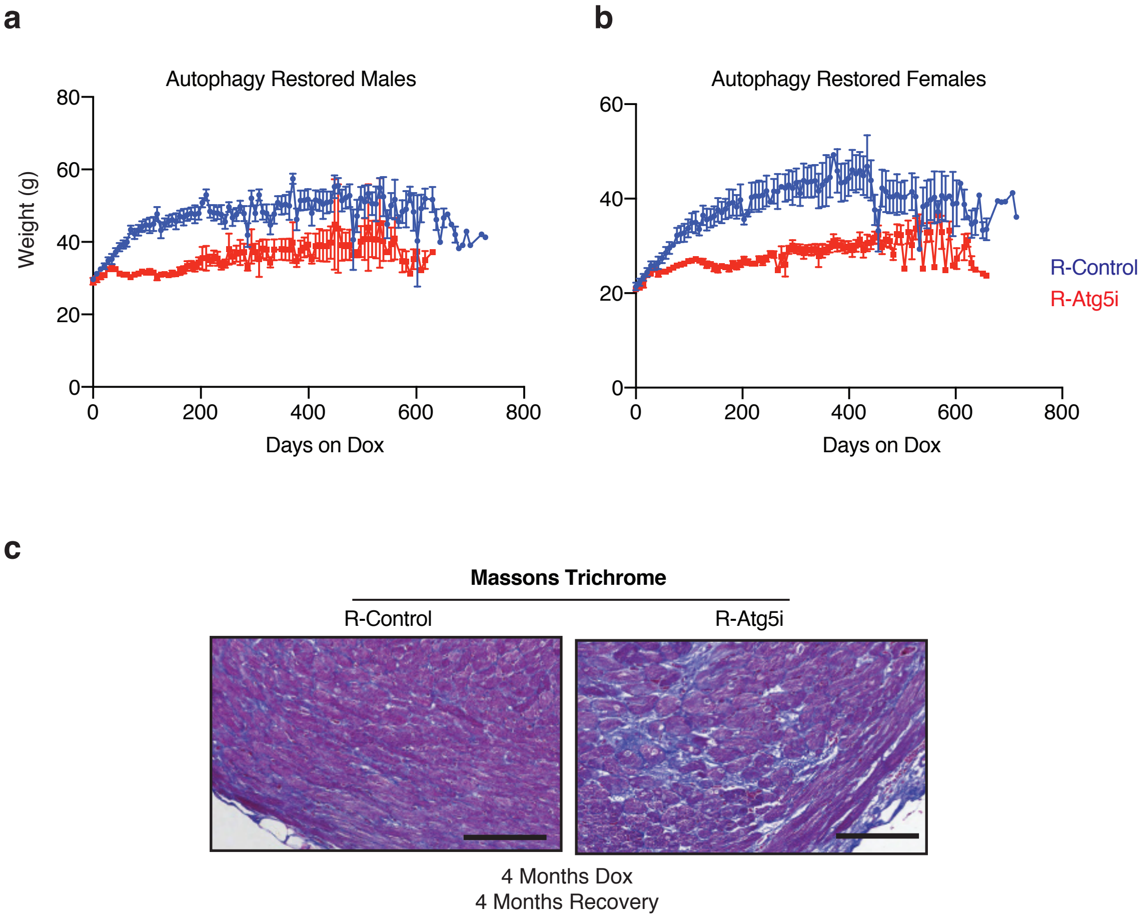

Supplementary Figure 9: Autophagy restoration displays segmental rescue of tissue phenotypes
a-**b**, Weights of mice from the R-Cohorts. **c**, Cardiac fibrosis was still present in R-Atg5i mice 4 months post dox removal.
