# Supplementary material for: Temporal inhibition of autophagy reveals segmental reversal of ageing with increased cancer risk
Source: Nat Commun. 2020 Jan 16;11:307. doi: 10.1038/s41467-019-14187-x (PMC6965206; doi:10.1038/s41467-019-14187-x)
Supplement: Supplementary file 3 — Description of Additional Supplementary Files [file 41467_2019_14187_MOESM3_ESM.pdf]

Description of Additional Supplementary Files

File type: Supplementary Movie. 1:

Description: R-Atg5i mice 4 months post dox removal highlighting the stochastic response to autophagy restoration. All mice were treated on dox for 4 months before dox removal for 2 months. At this stage, 100% of mice show kyphosis. The movie represents three examples with different levels of recovery. Mouse I exhibits little recovery, whereas mouse III looks normal with no sign of kyphosis. Mouse II appears active but with mild kyphosis.
