# Supplementary material for: Temporal inhibition of autophagy reveals segmental reversal of ageing with increased cancer risk
Source: Nat Commun. 2020 Jan 16;11:307. doi: 10.1038/s41467-019-14187-x (PMC6965206; doi:10.1038/s41467-019-14187-x)
Supplement: Supplementary file 5 — Reporting Summary [file 41467_2019_14187_MOESM5_ESM.pdf]

nature research

Corresponding author(s): Masashi Narita

Last updated by author(s): Oct 2, 2019

Reporting Summary

Nature Research wishes to improve the reproducibility of the work that we publish. This form provides structure for consistency and transparency in reporting. For further information on Nature Research policies, see Authors & Referees and the Editorial Policy Checklist .

Statistics

For all statistical analyses, confirm that the following items are present in the figure legend, table legend, main text, or Methods section.

n/a	Confirmed	
☐	☒	The exact sample size (*n*) for each experimental group/condition, given as a discrete number and unit of measurement
☐	☒	A statement on whether measurements were taken from distinct samples or whether the same sample was measured repeatedly
☐	☒	The statistical test(s) used AND whether they are one- or two-sided<br>*Only common tests should be described solely by name; describe more complex techniques in the Methods section.*
☒	☐	A description of all covariates tested
☒	☐	A description of any assumptions or corrections, such as tests of normality and adjustment for multiple comparisons
☐	☒	A full description of the statistical parameters including central tendency (e.g. means) or other basic estimates (e.g. regression coefficient) AND variation (e.g. standard deviation) or associated estimates of uncertainty (e.g. confidence intervals)
☐	☒	For null hypothesis testing, the test statistic (e.g. *F*, *t*, *r*) with confidence intervals, effect sizes, degrees of freedom and *P* value noted<br>*Give P values as exact values whenever suitable.*
☒	☐	For Bayesian analysis, information on the choice of priors and Markov chain Monte Carlo settings
☒	☐	For hierarchical and complex designs, identification of the appropriate level for tests and full reporting of outcomes
☒	☐	Estimates of effect sizes (e.g. Cohen's *d*, Pearson's *r*), indicating how they were calculated

Our web collection on statistics for biologists contains articles on many of the points above.

Software and code

Policy information about availability of computer code

Data collection	The open source software Fiji was used to segment and calculate muscle structure parameters<br>For kidney glomeruli sizing ImageScopeTM (Leica Biosystems) was used on pre-scanned slides<br>For immune quantification whole tissue sections were analysed using ImageScopeTM (Leica Biosystems)
Data analysis	Graphpad Prism: Version 7 was used for graph generation and statistical comparisons

For manuscripts utilizing custom algorithms or software that are central to the research but not yet described in published literature, software must be made available to editors/reviewers. We strongly encourage code deposition in a community repository (e.g. GitHub). See the Nature Research guidelines for submitting code & software for further information.

Data

Policy information about availability of data

All manuscripts must include a data availability statement. This statement should provide the following information, where applicable:

- Accession codes, unique identifiers, or web links for publicly available datasets
- A list of figures that have associated raw data
- A description of any restrictions on data availability

Data supporting the findings of this study are available within the article and its Supplementary Information files or from the corresponding author upon reasonable request

Field-specific reporting

Please select the one below that is the best fit for your research. If you are not sure, read the appropriate sections before making your selection.

Life sciences study design

All studies must disclose on these points even when the disclosure is negative.

Sample size	We note that no power calculations were used in this study.
Data exclusions	No data were excluded from analysis
Replication	All attempts at replication were successful
Randomization	Experimental mice and littermate controls were randomly assigned to time point assays or life-span assays together in such a manner that each experiment had paired experimental and littermate control mice.
Blinding	Investigators were blinded to the allocation during data collection and analysis with mice only referred to by their unique identification number until statistical tests were performed.

Reporting for specific materials, systems and methods

We require information from authors about some types of materials, experimental systems and methods used in many studies. Here, indicate whether each material, system or method listed is relevant to your study. If you are not sure if a list item applies to your research, read the appropriate section before selecting a response.

Materials & experimental systems

n/a	Involved in the study	
☐	☒	Antibodies
☒	☐	Eukaryotic cell lines
☒	☐	Palaeontology
☐	☒	Animals and other organisms
☒	☐	Human research participants
☒	☐	Clinical data

Methods

n/a	Involved in the study	
☒	☐	ChIP-seq
☒	☐	Flow cytometry
☒	☐	MRI-based neuroimaging

Antibodies

Antibodies used

All antibodies and dilutions are also described in the methods:
For Tissue staining:
Anti-P21 (Santa Cruz, SC-6246; 1:500)
Anti-TOM20 (Santa Cruz SC-11415, 1:500)
Anti-SQSTM1 (Enzo, BML-PW9860; 1:750)
Anti-KI67 (Bathyl Laboratories, IHC-00375; 1:1000)
Anti-LC3 (Nanotools, LC3-5F10 0231-100, 1:400)
Anti-γ-H2A.X (Cell Signalling Technologies, S139; 1:250)
Cy-3-labelled telomere specific (CCCTAA) peptide nuclei acid probe (Panagene)
Anti-dystrophin (Abcam, ab15277, 1:1000)
Anti-PAX7 (Abcam, ab34360, 1:1000)
Anti-LAMININ (Abcam, ab11576, 1:1000)
Anti-CD45-B220 (R&D Systems, MAB1217, 0.67ug/ml)
Anti-CD3 (Dako, A0452, 1:1000)
Anti-F4/80 (Serotec, MCA497, 1:20)

For western:
Anti-ATG5 (Abcam, ab108327; 1:1000)
Anti-LC3 (Abcam, ab192890; 1:1000)
Anti-ACTIN (Santa Cruz Biotechnology, I-19; 1:5000 [no longer commercially available])
Anti-P53 (Cell Signalling Technologies, Clone 1C12; 1:1000)
Anti-P21 (Santa Cruz, SC-6246; 1:1000)
Anti-Histone H3 (Abcam, ab1791, 1:5000)
Anti-P16 (Santa Cruz, SC-1207; 1:1000)
Anti-HMGA1 (Abcam, ab129153; 1:1000)
Anti-NBR1 (abcam, ab55474; 1:1000)

Validation

Antibody validation data is available on the manufacturers websites

Animals and other organisms

Policy information about studies involving animals; ARRIVE guidelines recommended for reporting animal research

Laboratory animals	The generation of mice containing an shRNA in a miR-E design was performed by Mirimus. Characterisation of the systemically inducible Atg5i mice are described in manuscript reference # (shRNA guide sequence from Mirimus Atg5_1065: TATGAAGAAAGTTATCTGGGTA). Atg5i_2 mice use a different shRNA guide sequence from Mirimus Atg5_1654: TTATTTAAAAATCTCTCACTGT. All mice are maintained on a mixed but uniform C57BL/6J and 129 background with littermate controls used in all experiments. The age, sex and treatment regimen combinations are as follows:<br>Long-term continuous doxycycline addition (LT-Cohorts) for survival analysis:<br>Atg5i Male n= 16 mice (none censored)<br>Control Male n= 16 (9 events censored)<br>Atg5i Female n=12 mice (none censored)<br>Control Female n= 19 (13 events censored)<br>Note that some control mice were culled to generate age-matched littermate control tissue. These numbers are shown in brackets here and are marked as censored events on the survival curve. For analysis mice were treated as alive up to the point of their removal from the study where they are considered lost to follow-up.<br><br>Four month doxycycline addition and then removal (R-Cohorts) for survival analysis:<br>Atg5i Male n= 8<br>Control Male n= 14<br>Atg5i Female n=14<br>Control Female n= 8
Wild animals	N/A
Field-collected samples	N/A
Ethics oversight	All experiments were performed in accordance with UK Home Office regulations and within national and institutional guidelines. Additionally the ethics review committee of the University of Cambridge approved this study.

Note that full information on the approval of the study protocol must also be provided in the manuscript.
