# Supplementary material for: Temporal inhibition of autophagy reveals segmental reversal of ageing with increased cancer risk
Source: Nat Commun. 2020 Jan 16;11:307. doi: 10.1038/s41467-019-14187-x (PMC6965206; doi:10.1038/s41467-019-14187-x)

Fig 3a and b:LC3, atg5. p21 and p53, blot loading control

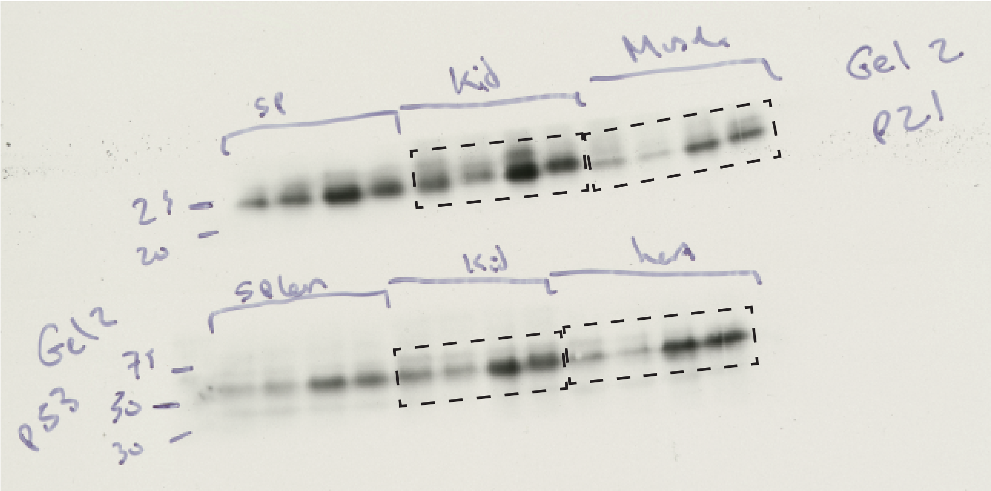

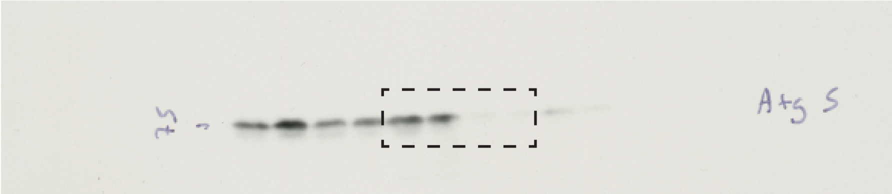

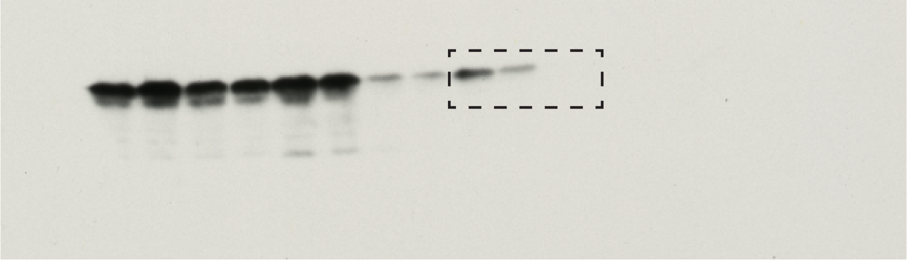

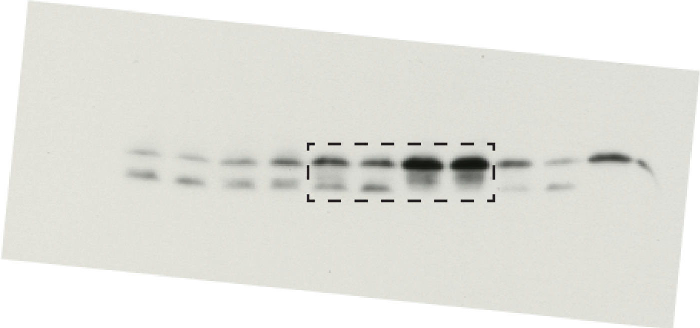

Lc3

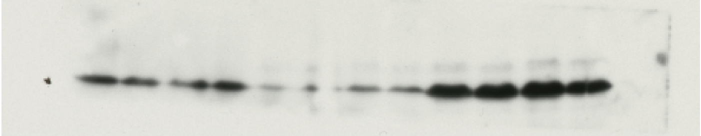

T-Actin

Fig 3a and b LC3, representative total actin

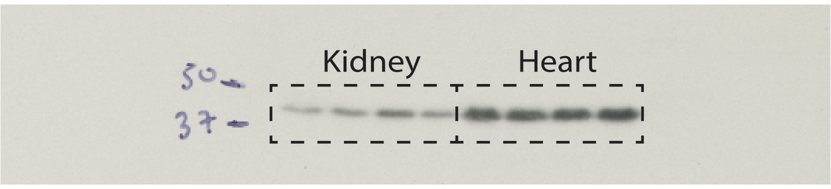

T-Actin loading control

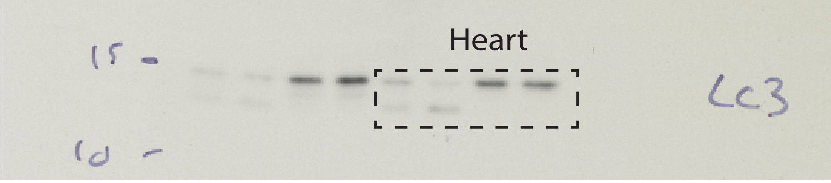

Lc3

Fig 3c: p16, blot loading control, Atg5

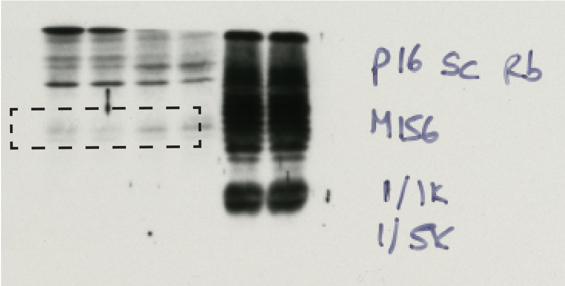

T-Actin loading control

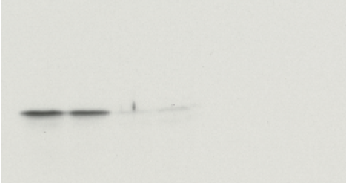

Atg5

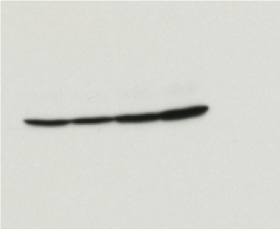

Fig 3c: p53, p21, blot loading control

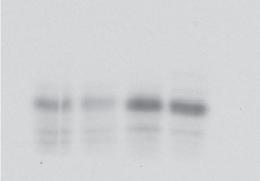

p53

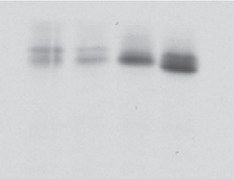

p21

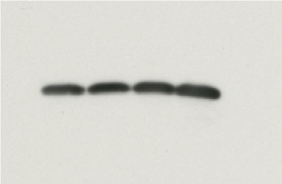

T-Actin loading control

Liver fig4

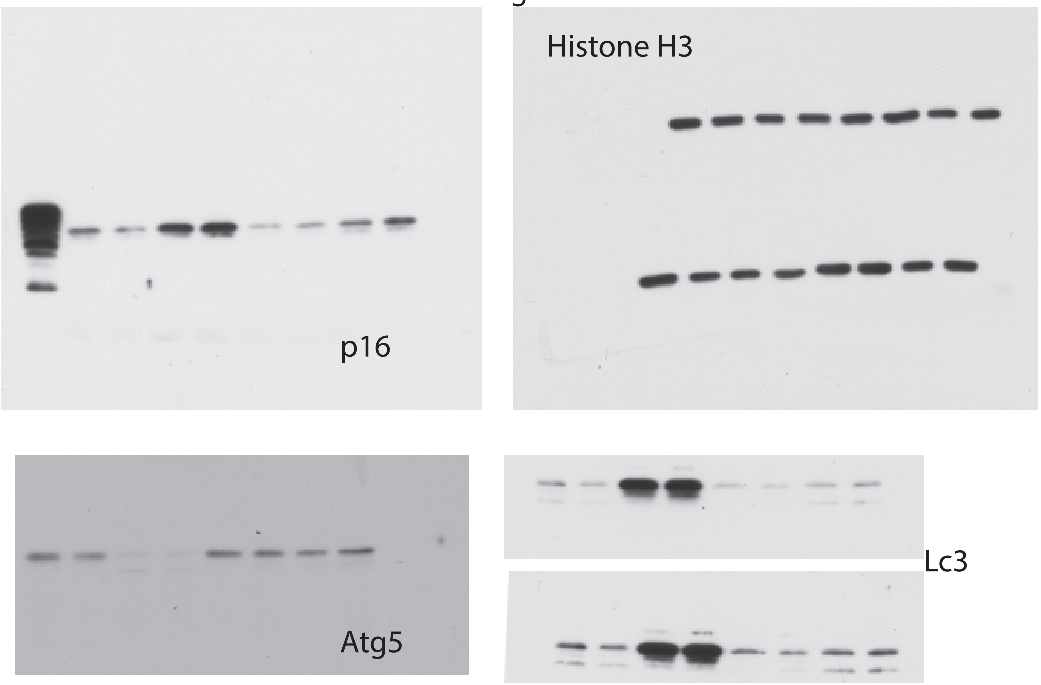

Kidney fig4

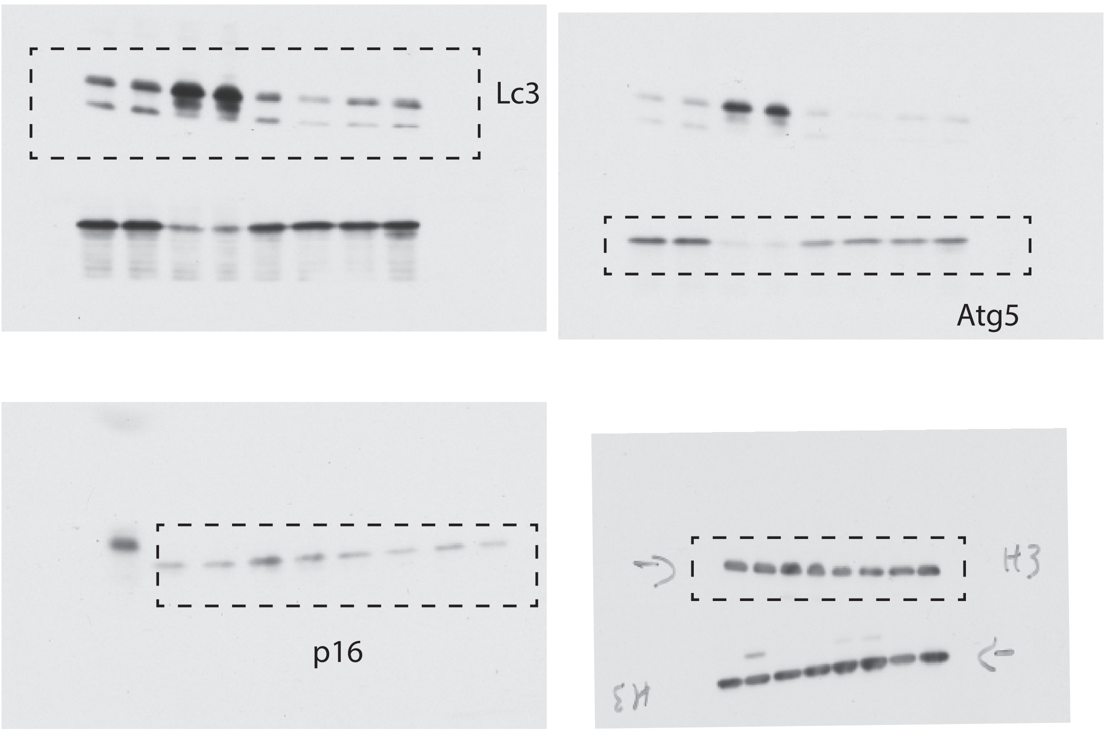

Supplementary Fig 1b: p16, blot loading control, Atg5

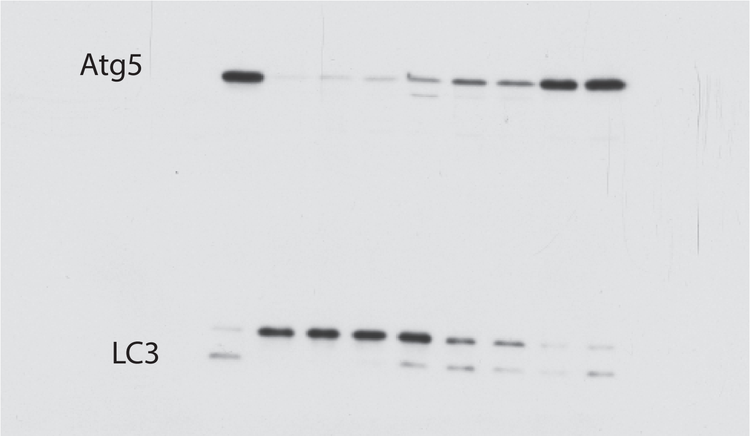

H3

NBR1

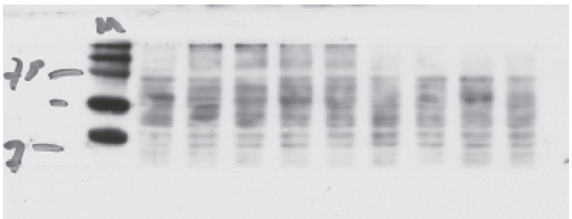

p62

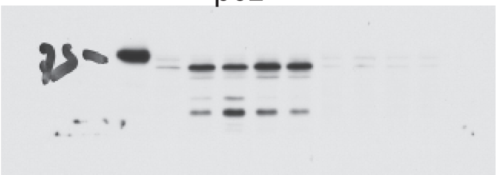

Supplemental Fig 6C : p16, p21 and p53, blot loading control

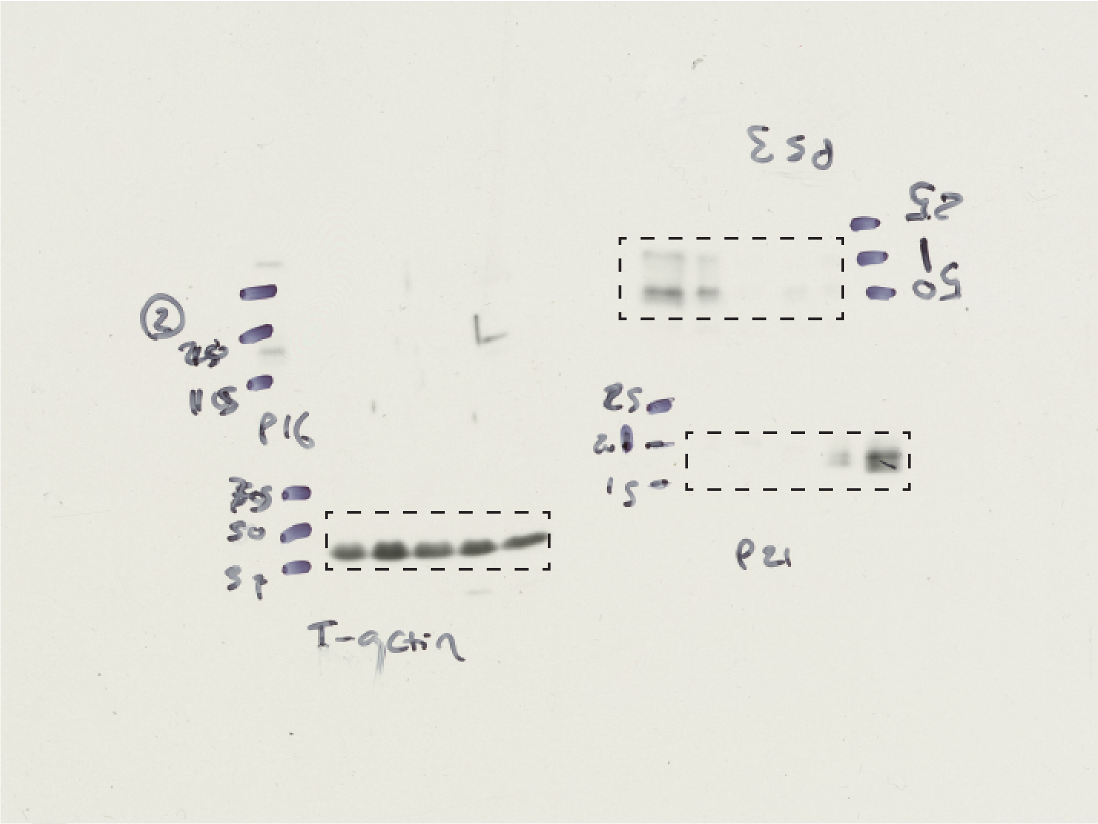

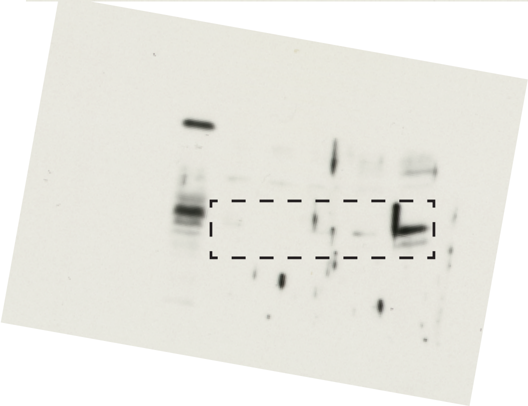

p16

Supplemental Fig 8a : atg5, p21 and lc3, blot loading control

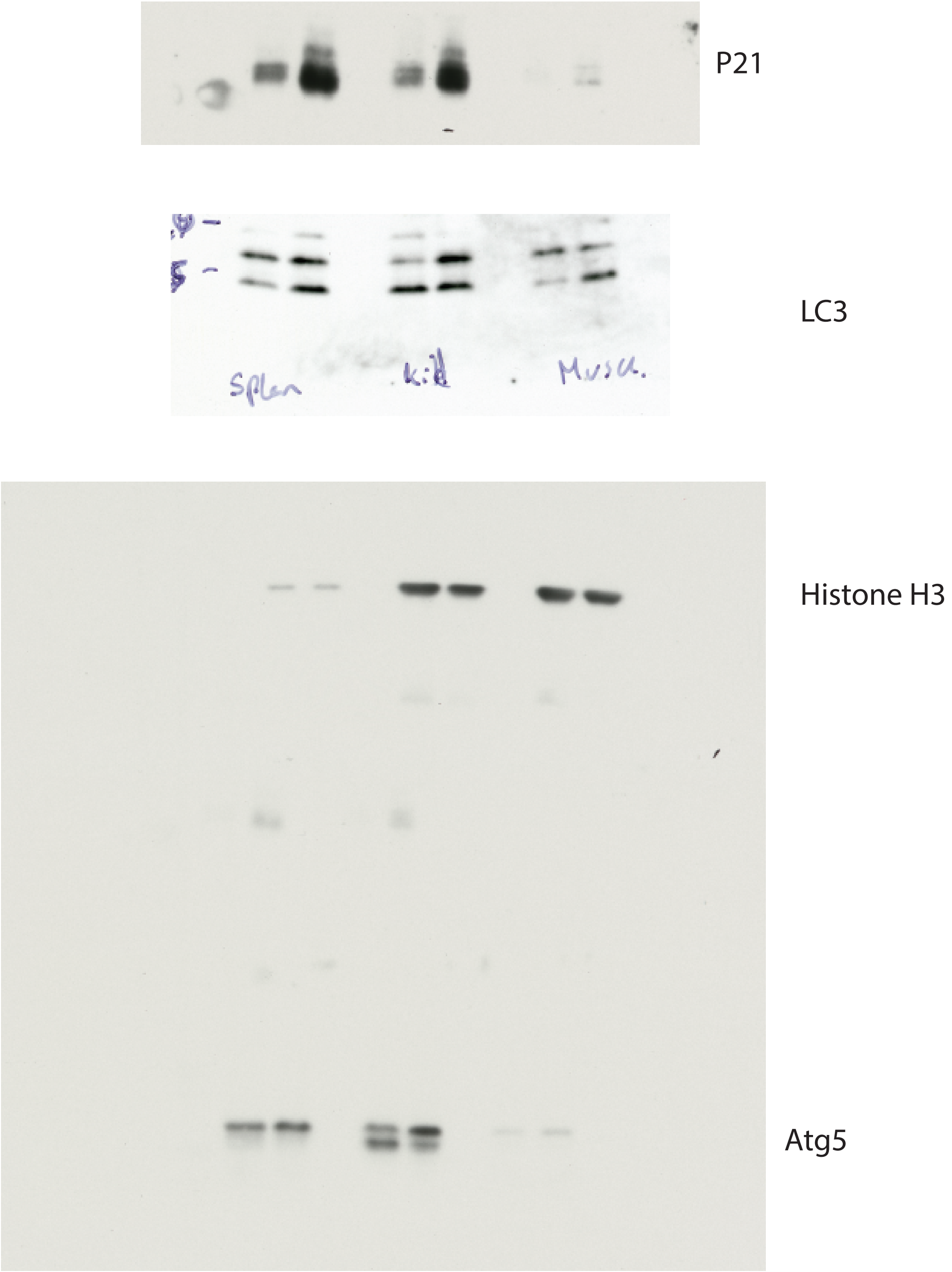

Supplement: Supplementary file 6 — Source Data [file 41467_2019_14187_MOESM6_ESM.pdf]
